# Cellular Characterization Using Microwave‐Assisted Immunocytochemistry, A Time‐Efficient Alternative to Traditional Immunocytochemistry

**DOI:** 10.1002/cpz1.70363

**Published:** 2026-04-13

**Authors:** Aidyn M. Medina‐López, Irma I. Torres‐Vázquez, Noraida Martínez‐Rivera, Eduardo Rosa‐Molinar

**Affiliations:** ^1^ Ronawk, Inc. Overland Park KS USA; ^2^ College of Natural Sciences, Department of Biology University of Puerto Rico at Rio Piedras San Juan PR USA; ^3^ Microscopy and Analytical Imaging Laboratory University of Kansas Lawrence KS USA; ^4^ Washington University center for cellular Imaging and Departments of Cell Biology Physiology, and Neuroscience, Washington University School of Medicine St. Louis MO USA; ^5^ This work was done to fulfill the requirements of a doctorate. (Medina‐Lopez, A. (2022). *Cell death in spinal cord injury: Which, where, when, and how* (Doctoral dissertation, University of Kansas). ProQuest Dissertations & Theses Global. https://www.proquest.com/openview/3244619b36c1015545280846f480418d/1.pdf?pq‐origsite=gscholar&cbl=18750&diss=y)

**Keywords:** immunocytochemistry, microwave‐assisted staining, blood‐spinal cord barrier (BSCB), neurovascular unit, cell characterization

## Abstract

Immunocytochemistry (ICC) and immunohistochemistry (IHC) are related but separate techniques used for cell and tissue characterization. ICC involves immunolabeling of cells, and IHC immunolabeling of tissues; thus, the main difference is sample thickness. However, the distinction between ICC and IHC is not always clear and can be confusing. We show that a clear distinction should be made between the two. Importantly, we show that assisted ICC is an excellent method for characterizing cell lines. It is quick, reduces background noise without disrupting the expression and organization of the protein of interest, and helps ensure the rigor and reproducibility of results. © 2026 The Author(s). Current Protocols published by Wiley Periodicals LLC.

**Basic Protocol 1**: Culture of blood‐spinal cord barrier and neurovascular unit cellular components

**Basic Protocol 2**: Immunocytochemistry assisted by PELCO BioWave® Pro (Fig. 3)

**Alternate Protocol**: Conventional immunocytochemistry

## Introduction

**Figure 1:**
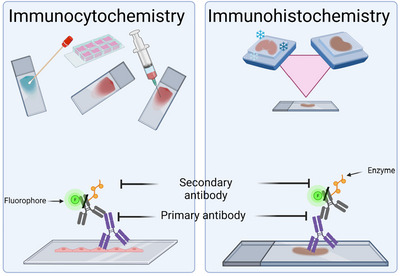
**Differences between Immunocytochemistry (ICC) and Immunohistochemistry (IHC)**. (A) ICC uses immunostaining and immunolabeling of cells that can originate from cell culture, primary cells, smears, swabs, or aspirates. (B) IHC involves immunostaining and labeling of whole‐mount tissues or tissue sections, either embedded in tissue blocks or frozen.

Antibodies’ unique properties of specificity, binding ability, and binding strength make them a critical tool for developing techniques such as immunoassays (Heitzmann & Richards, 1974; Guesdon, Ternynck, & Avrameas, 1979; Hsu et al., 1981; Malatesta, 2016; Renshaw, 2013). Immunocytochemistry (ICC) and Immunohistochemistry (IHC) are two of many immune assays. ICC is a technique that uses immunostaining and/or immunolabeling in cells derived from cultured cell lines or primary cells, such as those obtained from smears, swabs, and aspirates (Geethamala et al., 2017; Gillett, 2006; Malatesta, 2016; Renshaw, 2013; Zheng, 1998). IHC uses immunostaining and/or immunolabeling on whole‐mount tissue or tissue sections from paraffin‐ or plastic‐embedded or frozen tissue blocks. Thus, the primary difference is thickness [Figure 1]. However, ICC and IHC are often confused, and what is essentially an ICC is often reported as an IHC.

**Figure 2:**
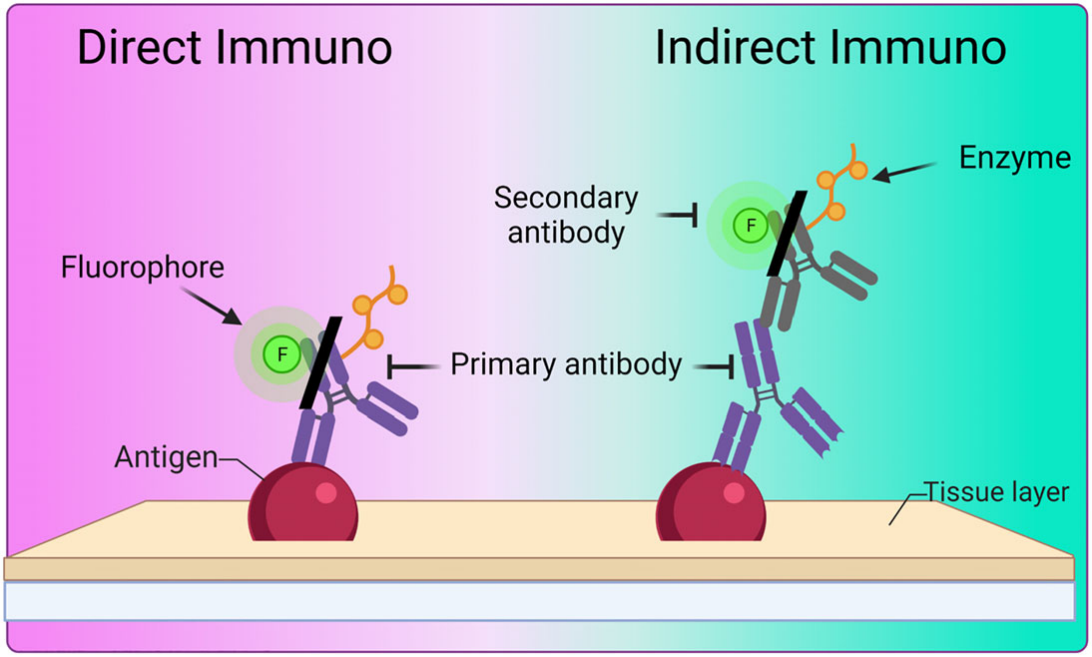
**Differences between direct and indirect ICC/IHC**. (A) Direct ICC/IHC involves the primary antibody conjugated to an enzyme or fluorophore. (B) Indirect ICC/IHC involves using a secondary antibody that is conjugated to an enzyme or a fluorophore.

Even though IHC and ICC differ, the principles are the same: an antibody is used to specifically bind to the protein of interest, also known as the primary antibody (Gillett, 2006; Malatesta, 2016; Renshaw, 2013). A reporter is then used to amplify the visualization of the primary antibody (Gillett, 2006; Malatesta, 2016; Renshaw, 2013). Both techniques can be performed either directly or indirectly (Gillett, 2006; Malatesta, 2016; Renshaw, 2013). The direct technique involves conjugating an enzyme or fluorophore directly to the primary antibody; the downside is the low signal amplification (Gillett, 2006; Malatesta, 2016; Renshaw, 2013). The indirect technique uses a secondary antibody specific to the primary antibody's species; the secondary antibody is conjugated to an enzyme or fluorophore (Gillett, 2006; Malatesta, 2016; Renshaw, 2013). This allows for greater signal amplification through multiple secondary antibodies binding to the primary [Figure 2] (Gillett, 2006; Malatesta, 2016; Renshaw, 2013). Using these definitions, we can identify relevant applications. IHC is a technique mainly used in pathology and clinical diagnosis. In contrast, ICC is commonly used in molecular biology fields (e.g., neuroscience, cell biology, drug discovery) mainly due to the higher resolution that a “thin slice” of cells provides. Nevertheless, the goal of both techniques is the same: to deliver qualitative and semi‐quantitative data in (1) protein expression and (2) localization within (a) individual cells (monoculture/ICC) or (b) multiple cells (co‐cultures/ICC, tissues/IHC) to allow visualization of spatial relationships and morphological structures.

Our work focused on indirect ICC. Even though ICC involves a low‐thickness sample, the entire procedure can take several hours to a day to complete (Kumada et al., 2004; Muñoz et al., 2004). The incubation time of the primary antibody (which depends on the target protein of interest) is a challenging and limiting factor in ICC. Too little incubation can lead to no binding, resulting in no/low signal (also a false negative); incubation that takes too long can lead to nonspecific binding (false positive). Several methods are used to simultaneously reduce incubation time and ensure labeling (note: labeling is limited by the presence of the protein of interest in the sample, the quality of the primary antibody, and the ability to visualize the reporter), but unlike other immunoassays such as western blotting or flow cytometry, which can quantify protein abundance and presence both of those techniques lose the spatial information we gain from both ICC and IHC. The preservation of the structural context of cells and other tissues at the expense of longer processing times and more labor‐intensive workflows is what makes these two techniques (ICC/IHC) among the most widely used in research.

Microwaves have been used to reduce the amount of time required for ICC and IHC (Kumada et al., 2004; Muñoz et al., 2004; Login & Dvorak, 1988). Leong and Milios introduced the use of microwaves in immunoassays in 1986 (Leong & Milios, 1990), and subsequent modifications to the original protocol and applications have been published since (a more detailed history of the use of microwaves can be found in Muñoz et al., 2004). Microwaves have been used primarily for processing electron microscopy (EM) samples (Leong & Milios, 1990; Login & Dvorak, 1988). However, advances in microwave instrumentation, including improved control of power output and heat regulation, have expanded their use to ICC and IHC applications. Microwave‐assisted labeling relies on controlled dielectric heating, which increases molecular motion in aqueous buffers by inducing dipole rotation in polar molecules (Boon & Kok, 1994; Giberson & Demaree, 1995). This controlled energy transfer can transiently increase antibody diffusion rates, facilitating faster antibody–antigen interactions and improving reagent penetration into cellular structures (Chicoine & Webster, 1998; Muñoz et al., 2004). In addition, the shorter incubation times associated with microwave‐assisted protocols reduce the opportunity for nonspecific binding, thereby improving signal‐to‐noise ratios (Sanders et al., 2006). When power and temperature are carefully regulated, microwave irradiation can enhance reaction kinetics without exceeding protein denaturation thresholds, preserving antigen structure and fluorophore stability (Giberson, 2002; Smith et al., 2008). As with traditional methods, microwave‐assisted ICC and IHC rely on the use of primary antibodies (monoclonal or polyclonal; see Lipman et al., 2005 for antibody classification) to label proteins of interest (Kumada et al., 2004; Leong & Milios, 1990; Lipman et al., 2005; Muñoz et al., 2004; Login & Dvorak, 1988). However, microwave‐assisted protocols can significantly reduce incubation time and background staining (Kumada et al., 2004; Muñoz et al., 2004). In our work, we also used microwave irradiation to reduce the incubation time required for organelle stains such as the nuclear stain DAPI (4′,6‐diamidino‐2‐phenylindole) and the membrane stain WGA (wheat germ agglutinin). Microwave‐assisted ICC/IHC, therefore, offers an opportunity to reduce the time required to perform these techniques while achieving reliable, reproducible labeling results.

This document includes protocols for (1) the culture of blood–spinal cord barrier components, which outlines specialized media formulations, routine cell culture practices, and practical tips for preparing cells for imaging; (2) conventional ICC, which details required solutions, incubation times, and step‐wise procedures for indirect ICC; and (3) microwave‐assisted ICC, which provides analogous information to the conventional protocol, along with step‐by‐step instructions that specify microwave settings, timing, power/voltage parameters, and optional vacuum conditions to optimize staining efficiency. While the conventional ICC protocol remains a reliable standard for routine workflows, the microwave‐assisted protocol is preferable when rapid turnaround, reduced reagent consumption, or minimized background staining is desired. Overall, this paper presents a streamlined, easy‐to‐follow workflow for cell characterization using microwave‐assisted ICC, which reduces processing time and consumables compared with conventional ICC methods.

*NOTE*: All protocols involving animals must be reviewed and approved by the appropriate Animal Care and Use Committee and must follow regulations for the care and use of laboratory animals. Appropriate informed consent is necessary for obtaining and use of human study material.

## Basic Protocol 1 CULTURE OF BLOOD‐SPINAL CORD BARRIER AND NEUROVASCULAR UNIT CELLULAR COMPONENTS

This protocol outlines a streamlined, easy‐to‐follow workflow for the culture and expansion of blood–spinal cord barrier and neurovascular unit cellular components. Users will culture endothelial cells, pericytes, astrocytes, and motor neurons under standard mammalian tissue culture conditions, utilizing appropriate coating substrates and media formulations to support cell attachment and proliferation. The workflow emphasizes establishing low‐passage stocks, maintaining healthy cultures through controlled passaging, and generating defined cell numbers for downstream experimentation. When implemented properly, this protocol is expected to yield reproducible cell growth and viability across cell types, enabling consistent culture preparation for subsequent characterization or assay‐based applications.

### Materials

Mouse brain microvascular endothelial (mBMECs) [Angio‐Proteomie, cat. no. cAP‐M0002]
mBMEC Complete Medium (See recipe)
Mouse Brain Vascular Pericytes (mBVP) [ScienCell Research Laboratories, cat. no. M1200]
mBVP Complete Medium (See recipe)
Mouse Astrocytes type 1 (mAs) [American Type Culture Collection, cat. no. CRL‐2541, RRID CVCL_6379]
mMA Complete Medium (See recipe)
Mouse hybrid motor neuron (mMNs) [Cederlane/ CELLutions Biosystems Inc., cat. no. CLU140]
mMN Complete Medium (See recipe)
Deionized sterile water
Poly‐L‐Lysine 0.01% [Millipore, cat. no. A‐0050C]
Fibronectin [Sigma, cat. no. F0895‐2 mg]
Trypsin 0.25% EDTA [ThermoFisher, cat. no. 25200072]
Dimethylsulfoxide (DMSO)

8‐well glass slides (8‐W) [ibidi cells in focus, cat. no. 80827]
T75 Flasks [MilliPore Sigma, cat. no. TPP 90026]
CO_2_ incubator Shel‐Lab [Sheldon Manufacturing, Model SCO6AD]
Class II Type A2 Purifier Biological Safety Cabinet (BSC) [LabConco, cat. no. 343000110815]
Serological pipettes (2, 10, 5, 10, and 25 mL)
Micropipettes (0.5–10, 2–20, 20–200, 100–1000 µL)
Pipette controller
200‐µL pipette tips
1000‐µL pipette tips
0.1–10 µL pipette tips
Water Bath at 37°C
Centrifuge [VWR, cat. no. 76019‐132]
Hemocytometer [Bright‐Line, cat. no. 1492]
Aspirator
Trinocular Inverted Biological Microscope with Phase Contrast [AmScope, model IN300T‐FL) objectives 4× (plan Fluor NA 0.15), 10× (Olympus, A10PL, NA 0.25), 20× (Plan Fluor PH NA 0.50), and 40× (Plan Fluor NA 0.60)].

Remember to culture in accordance with standard mammalian tissue culture protocols and aseptic techniques in a biological safety cabinet (BSC).

All listed volumes are for cultures grown in T75 flasks.

1 For mBVP cells, coat culture flasks with 0.01% poly‐l‐Lysine for 30 min.
2 For mMNs, coat culture flasks with 10 µg fibronectin for 30 min.
3 Rinse the coated flasks once with deionized sterile water and let them dry in the incubator.
4 Culture BSCB (Blood Spinal Cord Barrier) components (e.g., mBMECs, mBVPs, mAs, mMNs) according to standard mammalian tissue culture protocols and sterile techniques in their corresponding media.
5 Create a low passage stock (i.e., passage 2–4) for all cell lines by culturing them in a T75 flask, feeding them according to supplier instructions until 70%–80% confluency is reached.
6 Once cells are at 70%–80% confluency, add 2 mL trypsin and “trypsinize” for 5 min at 37°C.
7 To neutralize the trypsin, add 3 mL normal medium and pipette to remove attached cells from the plastic.
8 Collect the neutralized trypsin and detached cells in a conical tube.
9 Centrifuge the mixture for five minutes at 1500 RPM or 420 RCF at room temperature.
10 Remove the supernatant.
11 Resuspend pellet with 1 ml normal medium.
12 Count cells using a hemocytometer.
13 To create a stock of cells, collect one million cells in 900 µL normal medium and 100 µL DMSO.
14 Store at −80°C for up to one week.
  For long‐term storage, transfer to liquid nitrogen.
15 To follow the experimental design below with the cells, in an ibidi 8‐well slide, seed 50,000 cells in 200 µL normal medium per well.
16 Let the cells grow for at least 16 h.
  It is expected that doubling will happen during this time, allowing the cells to reach a total of 100,000 cells per well.

## Basic Protocol 2 IMMUNOCYTOCHEMISTRY ASSISTED BY PELCO BioWave® Pro (Figure 3)

**Figure 3:**
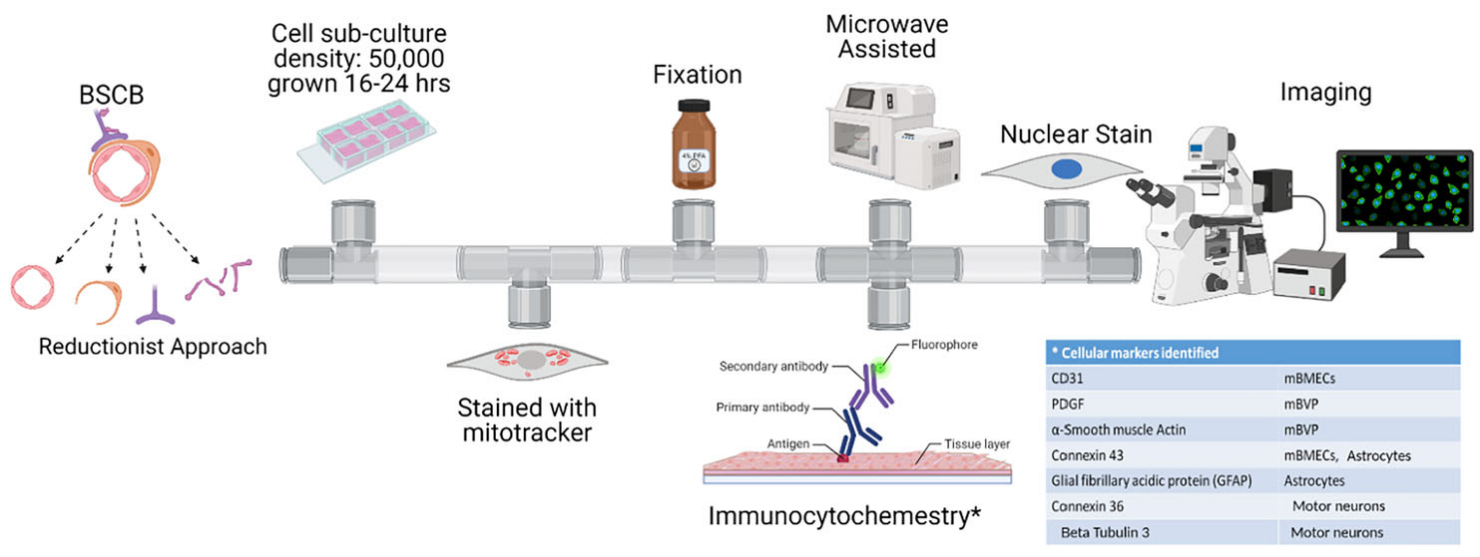
**Graphical schematic of microwave‐assisted immunocytochemistry**. Graphical depiction of the protocol to follow for microwave‐assisted ICC. This animated protocol also shows the labels/ proteins of interest selected for our targeted cell populations. Depending on the number of labels and stains used, this protocol can take from 20 min to 1.5 hr. Microwave‐assisted ICC is time‐ and cost‐effective compared with traditional ICC.

This protocol describes a streamlined workflow for performing immunocytochemistry using the PELCO BioWave® Pro, which leverages controlled microwave energy to accelerate reagent penetration and improve staining efficiency. Users will fix cells, perform sequential labeling with optimized buffer systems, and apply primary and secondary antibodies at defined dilutions and exposure parameters. By incorporating microwave‐assisted incubation, this workflow reduces overall processing time while maintaining antibody specificity and minimizing non‐specific background. When executed properly, the protocol should yield bright, uniform fluorescence labeling across target structures, enabling reliable visualization of cellular markers using widefield or confocal microscopy.

### Materials

8‐well glass slides (8‐W) [ibidi cells in focus, cat. no. 80827]
2% Paraformaldehyde (PFA) [16% aqueous Paraformaldehyde diluted in HBSS]
Hank's Balanced Salt Solution (HBSS) [ThermoFisher, cat. no. 14185‐052]
HBSS:Su (See recipe)
HBSS:Su:Sap (See recipe)
HBSS:Su:Sap:Tx (See recipe)
Blocking Solution Goat (See recipe)
Blocking Solution Donkey (See recipe)
Primary antibodies (see Table 1 for dilutions and target cells)
Secondary antibodies (see Table 2 for dilutions)
Cellular stains (see Table 3 for targeted organelles and dilutions).
Mounting medium, Vectashield [Vector Laboratories, cat. no. H‐1000‐10]

3i/Olympus Inverted Epifluorescent Microscope with ZDC [Olympus, model IX81]
Lambda LS Xenon [Sutter Instrument, cat. no. CO‐O661301]
Filter sets for 405, 488, 561, and 650 excitation wavelengths [Semrock, model Brightline® Sedat Filter Set for DAPI, FITC, TRITC, CY5, and CY7, Part no. 5 × 5M]
For the objective used, see the figure descriptions.
Zyla sCMOS Digital camera [Andor, model 4.2CL10]
3i/Olympus Inverted Spinning Disk Confocal [Olympus, model IX81]
Spinning Disk [Yokagawa, model CSU10]
CUBE 405 nm laser
Sapphire 488 nm laser
CrystaLaser 561 nm laser
CUBE 640 nm laser
Sutter Lambda 10‐3 filter wheel
Hamamatsu CMOS Flash Digital Camera [Hamamatsu, model 4.0 v1]
Pelco BioWave® Pro [Ted Pella, Inc., model Pelco BioWave® Pro 36500, Part no. 080‐300; Dec. 2006]
Analytical weight station
Conical tubes 15 mL
Microtubes (2 mL and 1.5 mL)
Macrotubes (5 mL)
Serological pipettes (2, 10, 5, 10, and 25 mL)
Micropipettes (0.5–10, 2–20, 20–200, 100–1000 µL)
Pipette controller
200 µL pipette tips
1000 µL pipette tips
0.1–10 µL pipette tips
Centrifuge [VWR, cat. no. 76019‐132]
Aspirator
SlideBook 6.0.21 (36497) ×64 Imaging Software [Intelligent, Imaging, Innovations, Inc (3i, Inc)].
BioRender [BioRender.com]
Photoshop [Adobe, Version 23.31]

**Table 1:** Primary antibodies for immunocytochemistry

Name	Cells target	Dilution	Company	Cat. No.	RRID
Rabbit anti‐Connexin 43	mBMECs, mAs	1:200	Sigma Aldrich	C6219	AB_476857
Rabbit anti‐CD‐31	mBMECs	1:200	ABclonal	A3181	AB_2764969
Rabbit anti‐alpha smooth muscle actin	mBVP	1:200	ABclonal	A17910	AB_2861755
Rabbit anti‐PDGFRB	mBVP	1:200	ABclonal	A19531	AB_2832987
Rabbit anti Connexin 36	mMNs	1:200	Zymed	36‐4600	AB_2533260
Rat anti‐Glial Fibrillary Acidic Protein	mAs	1:500	Calbiochem	345860	AB_10685458
Rabbit anti Beta Tubulin 3	mMNs	1:100	Sigma	T‐2200	AB_262133

**Table 2:** Secondary antibodies for immunocytochemistry

Name	Dilution	Company	Cat. No.
Goat anti‐Rabbit 647	1:200	Life Technologies	A21245
Donkey anti‐Rat 488	1:200	Life Technologies	A21208

**Table 3:** Reference cellular counterstains

Target organelle	Name	Live/ Fixed	Dilution	Company	Cat. No.
Mitochondria	Mitotracker C‐Rox	Live and Fixed	250 nM	Molecular Probes	M7512
Cell Membrane	Wheat Germ Agglutinin Alexa Fluor 488	Live and Fixed	5 µg/mL	Molecular Probes	W11261
Nuclear Stain	DAPI	Fixed	10 mM	Molecular probes	D1306

Following Burry (2011) and Hewitt et al. (2014), the PANC‐1, HeLa, and BJ cell lines served as positive and negative controls for immunocytochemistry. These cell lines were exposed to the same conditions as our cell lines of interest (mBMECs, mBVPs, mAs, mMNs), including, but not limited to, fixatives, antibody dilutions, buffers, and exposure times.

1 Culture cells in an 8‐well slide at a concentration of 50,000 cells per well with 200 µL normal medium per well
2 Fix slides with 2% PFA for ten minutes at room temperature.
3 Follow Table 4 PELCO BioWave® Pro assisted Immunocytochemistry protocol for solutions, times, and voltage information.

**Table 4:** PELCO BioWave® pro‐assisted immunocytochemistry steps

Step	User Prompt(on/off)	Time (hr:min:sec)	Power (Watts)	Temp (°C)	Vacuum/Bubble Pump (off/bub/vac cycle/vac on)	Load Cooler (off/auto/on)	Steady Temp pump (on/off)	Steady Temp Temp (°C)
1. Rinse with HBSS:Su	ON	00:01:00	150	80	OFF	AUTO	OFF	30
2. Blocking ON with 3% NS	ON	00:01:00	150	80	VACUM CYCLE	AUTO	OFF	30
3. Blocking OFF with 3% NS	OFF	00:01:00	0	80	VACUM CYCLE	AUTO	OFF	30
4. Blocking ON with 3% NS	OFF	00:01:00	150	80	VACUM CYCLE	AUTO	OFF	30
5. Rinse with HBSS:Su:Sap	ON	00:01:00	150	80	OFF	AUTO	OFF	30
6. 1^ry^ AB ON	ON	00:06:00	150	80	VACUM CYCLE	AUTO	OFF	30
7. 1^ry^ AB OFF	OFF	00:04:00	0	80	VACUM CYCLE	AUTO	OFF	30
8. 1^ry^ AB ON	OFF	00:06:00	150	80	VACUM CYCLE	AUTO	OFF	30
9. Rinse with HBSS:Su:Sap	ON	00:01:00	150	80	OFF	AUTO	OFF	30
10. Rinse with HBSS:Su:Sap	ON	00:01:00	150	80	OFF	AUTO	OFF	30
11. 2^ry^ AB ON	ON	00:06:00	150	80	VACUM CYCLE	AUTO	OFF	30
12. 2^ry^ AB OFF	OFF	00:03:00	0	80	VACUM CYCLE	AUTO	OFF	30
13. 2^ry^AB ON	OFF	00:06:00	150	80	VACUM CYCLE	AUTO	OFF	30
14. Rinse with HBSS:Su:Sap	ON	00:01:00	150	80	OFF	AUTO	OFF	30
15. Rinse with HBSS	ON	00:01:00	150	80	OFF	AUTO	OFF	30
16. Stain 1	ON	0:04:00	150	80	VACUM CYCLE	AUTO	OFF	30
17. Stain 1	OFF	0:03:00	0	80	VACUM CYCLE	AUTO	OFF	30
18. Stain 1	OFF	0:04:00	150	80	VACUM CYCLE	AUTO	OFF	30
19. Rinse with HBSS	ON	00:01:00	150	80	OFF	AUTO	OFF	30
20. Rinse with HBSS	ON	00:01:00	150	80	OFF	AUTO	OFF	30

4 Mount using Vectashield
5 Visualize in a Fluorescence scope.

## Alternate Protocol 1 CONVENTIONAL IMMUNOCYTOCHEMISTRY

This protocol provides a standard workflow for performing conventional immunocytochemistry on the blood–spinal cord barrier and neurovascular unit cell types. Users will fix, permeabilize, block, and sequentially label cells with primary and secondary antibodies under ambient conditions, using defined buffer systems and incubation times to support specific antibody binding. The workflow is performed on chamber slides without microwave assistance, relying instead on room‐temperature incubations to allow gradual reagent diffusion and protein interactions. When executed properly, this protocol should produce a clear, specific fluorescence signal with minimal background, enabling reliable visualization of cellular markers across multiple targets and cell types using widefield or confocal microscopy.

### [Additional] Materials

See Materials Listed in Basic Protocol 2. As the title implies, there would be no need to use the Pelco BioWave Pro.

Following Burry (2011) and Hewitt et al. (2014), the cell lines PANC‐1, HeLa, and BJs served as positive and negative controls for immunocytochemistry. These cell lines were exposed to the same conditions as our cell lines of interest (mBMECs, mBVPs, mAs, mMNs), including, but not limited to, fixatives, antibody dilutions, buffers, and exposure times.

1 Culture cells in an 8‐well slide at a concentration of 50,000 cells per well with 200 µl normal medium per well
  mBVP and mMNs need coating, poly‐L‐Lysine, and fibronectin, respectively.
2 Fix slides with 2% PFA for ten minutes at room temperature
3 Rinse three times with HBSS:Su for five minutes each at room temperature.
4 Permeabilize the cells three times with HBSS:Su:Sap for five minutes at room temperature.
5 Block the cells with 3% normal serum for thirty minutes at room temperature.
6 Rinse twice with HBSS:Su:Sap for five minutes each at room temperature.
7 Add primary antibody (Table 1) and incubate for 2 hr at room temperature or overnight at 4°C
8 Rinse three times with HBSS:Su:Sap for five minutes each at room temperature.
9 Add secondary antibody (Table 2) and incubate for one hour at room temperature.
  Before adding secondary, centrifuge the stock and the working dilution for 1 min at 16,000 rpm at room temperature; this will reduce the amount of unbound clumped secondary antibody in the preparation.
  Secondary incubation overnight is not recommended.
10 Rinse once with HBSS:Su:Sap for five minutes each at room temperature.
11 Rinse once with HBSS:Su for five minutes each at room temperature.
12 Rinse once with HBSS for five minutes at room temperature.
13 Add counterstains individually (Table 3) for thirty minutes at room temperature.
  Mitotracker is a live stain; this means cells are stained before fixation. Mitotracker is diluted in complete media and incubated for thirty minutes at 37°C.
14 Data shows stains and antibodies used.
15 Rinse three times with HBSS for five minutes each at room temperature.
16 Mount using Vectashield.
17 Visualize under a fluorescence microscope.

## Reagents and Solutions

Use distilled or deionized water for preparations as needed.

### Complete medium, mBMECs

**Table:** 

Reagent	Final concentration	Amount
DMEM	86% (v/v)	86 mL
FBS	10% (v/v)	10 mL
P/S	1% (v/v)	1 mL
2‐mercaptoethanol	1% (v/v)	1 ml
Non‐essential amino acids	1% (v/v)	1 mL
Sodium pyruvate	1% (v/v)	1 mL
Heparin	15 units/mL	Depends on batch
VEGF	5 ng/mL	Depends on batch
Total	100 mL	

### Complete medium, mBVPs

**Table:** 

Reagent	Final concentration	Amount
Basal pericyte media	89% (v/v)	89 mL
FBS	10% (v/v)	10 mL
P/S	1% (v/v)	1 mL
Pericyte growth supplement	1% (v/v)	1 mL
Total		100 mL

### Complete medium, mAs

**Table:** 

Reagent	Final concentration	Amount
DMEM	89% (v/v)	89 mL
FBS	10% (v/v)	10 mL
P/S	1% (v/v)	1 mL
Total		100 mL

### Complete medium, mMNs

**Table:** 

Reagent	Final concentration	Amount
DMEM	89% (v/v)	84 mL
FBS	10% (v/v)	10 mL
P/S	1% (v/v)	1 mL
Sodium bicarbonate	3.7 g/L	5 mL
Total		100 mL

### HBSS:Su buffer

**Table:** 

Reagent	Final concentration	Amount
10× HBSS	1×	5 mL
Sucrose	0.01% (w/v)	0.005 g
Distilled or Deionized Water	99.99%	45 mL
Total		50 mL

### Mitotracker labeling

**Table:** 

Reagent	Final concentration	Amount
Mitotracker CMX ROS	250 nM	0.5 µL
Complete Medium	99.975%	1,999.5 µL
Total		2 mL

### Hoechst labeling

**Table:** 

Reagent	Final concentration	Amount
Hoechst	5 µg/mL	0.8 µL
Complete Medium	99.96%	1999.2 µL
Total		2 mL

### Nuclear and mitochondrial live stain cocktail

**Table:** 

Reagent	Final concentration	Amount
Hoechst	5 µg/mL	0.8 µL
Mitotracker CMX ROS	250 nM	0.5 µL
Complete Medium	99.935%	1998.7 µL
Total		2 mL

### Permeabilization buffer

**Table:** 

Reagent	Final concentration	Amount
1× HBSS	1×	50 mL
Sucrose	0.01% (w/v)	0.005 g
Saponin	0.1% (w/v)	0.05 g
Total		50 mL

### Blocking buffer

**Table:** 

Reagent	Final concentration	Amount
Permeabilization Buffer	1×	9700 µL
Normal Serum	3% (v/v)	300 µL
Total		10 mL

### Primary antibody diluent buffer

**Table:** 

Reagent	Final concentration	Amount
Blocking Buffer	1×	9999 µL
Triton X	0.01%	1 µL
Total		10 mL

### Membrane labeling, post‐fix

**Table:** 

Reagent	Final concentration	Amount
1× HBSS:Su	1×	4975 µL
WGA stock	5 µg/mL	25 µL
Total		5 mL

### Membrane labeling, live

**Table:** 

Reagent	Final concentration	Amount
Complete medium	1×	4975 µL
WGA stock	5 µg/mL	25 µL
Total		5 mL

## Commentary

### Critical Parameters

#### Cell Culture

- The culture of cells in glass or optical‐optimized plastic is crucial for the visualization of samples after ICC.
  ○ This ensures optimal clarity, proper refractive index, minimized autofluorescence, and uniform flatness for easier focus.
- While glass is often preferred for imaging cells, it typically lacks the surface proteins necessary for proper cell attachment.
  ○ Tests should be performed to determine whether a specialized coating is needed to ensure proper cell adhesion.
  ○ Tip: always start with the basics, such as gelatin, collagen, and Poly‐L‐lysine. If none of these works, then move to more specialized coatings, such as Poly D‐Lysine, Poly ornithine, fibronectin, fibrinogen, laminin, vitronectin, and tumor‐derived extracellular matrix (ECM). A combination of any of these can also help diversify the attachment points for your cells.
- Seeding density of your slide depends on (1) your protein(s) of interest, (2) your schedule, and (3) the cells' doubling time (Abcam, 2024b).
  ○ Protein of interest: The expression of your protein/antigen of interest can be linked to specific cellular events. For example, proteins associated with tight junctions may not be highly expressed if cells are not at confluence. This means that if you would like to image these antigens, the cells need to reach confluency on your imaging slide before performing the ICC to ensure antibody labeling. This issue can be overcome by increasing your seeding density when preparing your cells, ensuring that, at the time of fixation, your cells are confluent and can be labeled with your antibody.
  ○ Schedule and doubling time: Always take your cells’ doubling time into consideration; it can affect your schedule and any decision to increase or decrease your seeding density for your experiment.

#### ICC

- **Fixation**
  We recommend 2% PFA for cells, as morphological stains were applied to live cells (e.g., Mitotracker CMX Ros for mitochondria). In previous runs, we observed that higher fixative concentrations interfered with the stain by (1) reducing its signal and (2) distorting its location (e.g., signal in both mitochondria and nearby organelles).
  The fixation time for a cellular sample, if no live stain is used, can be increased to 4% for up to 10 min.
  Over‐fixation can (1) increase the autofluorescence and overall noise of the sample, (2) obscure the antigen and then reduce the antibody accessibility to it, and (3) alter the spatial architecture and morphology of the cells. (Shi, Shi & Taylor, 2011, Fox et al., 1985, Schnell et al., 2012)
- **Buffers**
  All buffers in this protocol contain sucrose, which regulates sample osmolarity and tenacity, improving access to antigens and overall tissue/cell integrity.
- **Permeabilization**
  In this paper, we utilized two permeabilization buffers: (a) HBSS:Su:Sap and (b) HBSS:Su:Sap:TX (also described as Primary antibody diluent).
  Buffer A is a gentle buffer that prevents significant membrane disruption and false results. This buffer is used for short periods and repeated multiple times to ensure even, controlled membrane permeabilization.
  Buffer B has the same components as A, plus the stronger detergent TritonX. We also call it the “primary antibody diluent.” This buffer ensures that the primary antibody can access all parts of the cell, both the cell surface and intracellular compartments. In this paper, its use is limited to incubating the primary antibody; like buffer A, it serves as a control and promotes even membrane permeabilization during incubation (Abcam, 2024b).
  ○ Buffer selection depends on the antigen of interest.
**Extracellular/Surface antigens**: If your antigen of interest is on the cell surface or in the extracellular space, Buffer A is best.
    Your primary antibody must recognize the extracellular portion of your protein/antigen of interest; if not, additional permeabilization or other methods to expose the antigen (e.g., antigen retrieval) may be needed.
**Intracellular antigens**: If your antigen of interest is located in the intracellular compartments of the cells, potentially enclosed by multiple layers of membrane, buffer B is recommended.
    Since buffer B contains a stronger detergent that permeabilizes the main cell membrane and other organelle membranes, it will not be a challenge.
    While this might be the best for your needs, consider multiple exposures at shorter intervals to ensure even exposure and control permeabilization.
- **Blocking Buffer and Antibodies**
  For indirect ICC, it is key that we identify the host in which our antibodies were raised to properly select our normal serum for the blocking buffer. As defined in the Reagents and Solutions, our blocking buffer consists of 97% permeabilization buffer and 3% normal serum. The species of the serum selected is always the species in which the secondary antibody was raised/developed. This information is always available on the manufacturer's website or certificate of analysis.
  Example: The host in which the secondary antibody was raised/developed is a goat
  Secondary: Goat anti‐mouse AlexaFluor 488
  Normal serum in blocking buffer: Normal Goat Serum
  Indirect ICC requires two antibodies: the primary, which recognizes the protein/antigen of interest, and the secondary, which recognizes the primary (Abcam, 2024b). When selecting the primary and secondary antibodies, it is important to consider (1) your cell species (e.g., human, mouse, rat, etc.); (2) the reactivity of the primary antibody (in which species it has been recorded that the antibodies can identify the antigen/protein of interest); (3) the primary host species; (4) the primary type of immune globulin; (5) the secondary antibody target and (6) the secondary antibody host.
  Examples:
  Cell species: If none of the antibody hosts (both primaries and secondaries) are the same as the cell species, the results will yield false positives, as they may bind to the cells rather than the antigen of interest.
  Primary antibody reactivity: If the manufacturer lists the species that the antibody reacts with and your species is not among them, this could cause a false negative result, as the antibody will not bind and no signal will be observed (Abcam, 2024b).
  Primary host species: Could lead to false‐positive labeling if it matches the cell species.
- **The following information is critical for selecting your secondary antibody**:
  Your primary immunoglobulin type: There are several types of immunoglobulins (e.g., IgG, IgM, IgA, IgG1, IgG1b, etc.). If your primary is one of these “obscure” immunoglobulins, your secondary target needs to be that type of immunoglobulin; otherwise, you'll get a false negative result as well, since the immunoglobulins don't match.
  Your secondary antibody target: This includes the species and, as mentioned above, the immunoglobulin type.
  Example: Your primary was raised in rabbit, and it is an IgG2; your secondary needs to be Host anti‐rabbit IgG2 (remember the host can be any species other than your cell's species or the primary host species).
  Your secondary antibody host: This cannot match your cell species nor your primary host species. This is especially true if more than one antigen of interest is to be probed in the same sample and no serial ICC is to be performed. Both hosts must be different.
- **Controls**
  Positive and negative controls to characterize your primary antibody are critically needed. These can be tissues known to express your protein of interest, as well as tissues that do not. If these are not accessible modified cells that overexpress the antigen, or if the antigen is knock‐out (KO), then these cells can also serve as controls.
  Controls for your secondary are also needed.
  For more information on all the controls that should be performed, please see Hewitt et al., 2014
- **Organelle Staining**
  While organellar staining might not be needed, it is strongly recommended. Morphology staining is a landmark that can help post‐analyze your sample and serve as a reference for your labels, indicating exactly where they are located. Is there a relationship between your antigen of interest and this organelle? Why do you need landmarks?
  Some tips and tricks for these include ensuring that those strains can be fixed or used in fixed cells. The stains presented in this protocol are those that need to be used while the cell is alive (Mitotracker) and can be fixed afterwards. And stains that can be used in fixed cells (DAPI, Hoechst, WGA, etc.)
- **Mounting medium**
  Selecting a mounting medium is simple; its main goal is to protect the sample from fading. This can be a simple homemade recipe with glycerol or a commercially available product. (Platt & Michael, 1985)
- **Microwave**
  Microwave‐assisted immunocytochemistry enhances staining performance primarily through controlled dielectric heating, which increases molecular motion by inducing dipole rotation of polar molecules within aqueous buffer systems (Boon & Kok, 1994; Giberson & Demaree, 1995). This rapid, volumetric energy transfer transiently elevates the diffusion coefficient of antibodies, accelerating antibody–antigen encounter rates and improving reagent penetration into cellular compartments (Chicoine & Webster, 1998; Muñoz et al., 2004). Increased molecular mobility reduces boundary‐layer diffusion limitations and promotes more uniform reagent distribution across the specimen (Galvez et al., 2006). Importantly, the shortened incubation time associated with microwave ON/OFF cycling decreases the temporal window for nonspecific binding, thereby improving signal‐to‐noise ratios (Sanders et al., 2006; Muñoz et al., 2004). When power and temperature are tightly regulated, this controlled energy input enhances reaction kinetics without exceeding protein denaturation thresholds, preserving antigen integrity and fluorophore stability (Giberson, 2002; Smith et al., 2008). Thus, microwave‐assisted ICC improves labeling efficiency by accelerating diffusion and enhancing kinetics rather than by bulk thermal effects alone.
  There are several parameters to consider when using the microwave that, when controlled properly, can make your ICC a success. These include the control of power, temperature, irradiation uniformity, and cooling. Controlling these would also enhance the reproducibility and reliability of your samples (Boon & Kok, 1994; Giberson & Demaree, 1995). While the protocol of this manuscript has shown reproducibility across the selected cell lines and markers, we also have to take into consideration that this is due to the type of microwave used, the Ted Pella Bio‐wave Pro. Nevertheless, understanding the parameters already mentioned would help us apply that knowledge and troubleshoot any other microwave available.
- **Power**
  The power in a microwave determines the rate of energy transfer and diffusion. Control of the wattage is essential for maintaining antigen integrity and improving the signal‐to‐noise ratio (Giberson, 2002). Utilizing this to our advantage, low‐to‐moderate power improves antibody penetration and reaction kinetics, whereas high power tends to denature the antigens and even accelerate fluorophore degradation. (Galvez et al., 2006; Sanders et al., 2006)
  ○ The recommended Range for ICC is 100‐250 W
  ○ As established in Table 4, the power utilized in the data provided in this manuscript is between the ranges acceptable for a reproducible result.
- **Temperature**
  Temperature is a key parameter for epitope preservation and fluorescence stability. Most immunochemical reactions have a narrow temperature range for optimal results (Boon & Kok, 1994).
  For antibody incubation, the maximum recommended temperature is ≤37°C, but the ideal range is 20–30°C. As shown in Table 4, we did not deviate from 30°C in any condition.
  Temperatures exceeding 55°C cause irreversible protein denaturation, increased autofluorescence, and morphological distortion (Giberson & Demaree, 1995; Smith et al., 2008).
  For this reason, active temperature control during any microwave protocol is necessary. Recirculating cooling systems and temperature probes help prevent thermal spikes and maintain stable conditions (Giberson et al., 1997). Continuous monitoring ensures samples remain within the validated range, particularly during antibody incubation and fluorescence labeling, where excess heat can compromise signal quality (Muñoz et al., 2004).
- **Load Cooling and Field Homogenization**
  Microwaves can produce uneven heating within the cavity. Adding a water load or using a ColdSpot™ device helps absorb excess energy and improve heat distribution (Giberson & Demaree, 1995; Josephsen et al., 2005).
  Standardized systems, such as the ColdSpot™ platform integrated with the PELCO BioWave accessories (Smith et al., 2008), improve temperature uniformity, reduce localized overheating, and enhance run‐to‐run reproducibility (Muñoz et al., 2004).
- **Tissue and Culture Thickness**
  Although microwave irradiation enhances molecular diffusion, tissue thickness remains a limiting factor. Ultrathin and monolayer samples exhibit optimal labeling, whereas thick preparations show penetration gradients. (Chicoine & Webster, 1998; Muñoz et al., 2004)
  Multilayer cultures and 3D systems, therefore, require extended incubation and reduced power settings.
  This protocol also applies to complex cultures (e.g., multiple cells or co‐cultures) but extends the incubation time by 1–3 min, depending on culture thickness.
  This protocol is for monolayer cells; time and power can be modified for 3D in vitro systems (i.e., Organoids, spheroids, hydrogels).
  The versatility of this protocol has yielded advantageous results in culture volume [Torres‐Vasquez et al. (personal Communications)].
- **Buffers and Ionic Strength**
  Buffer composition strongly influences microwave absorption. High‐ionic‐strength buffers heat more rapidly and may generate localized thermal artifacts (Galvez et al., 2004; Hajibagheri, 1999).
  All buffers should be freshly prepared, filtered, and standardized across experiments.

### Troubleshooting

**Table:** 

Category	Problem	Possible cause	Diagnostic check	Solution	References
Signal Detection	No signal	Primary antibody not reactive to the species	Is it primarily validated for cell species?	Use species‐validated primary	Abcam, 2024b Burry, 2011 Hewitt et al., 2014 Lipman et al., 2005 Lee & Kitaoka, 2018 Giberson, 2002 Kumada et al., 2004 Muñoz et al., 2004 Boon & Kok, 1994
		Host species conflict	Does the primary host match the cell species?	Select a different host species	
		Secondary mismatch	Does secondary match species AND Ig subtype?	Use a correct secondary antibody	
		Low antigen expression	Are cells at the required confluency/state?	Adjust seeding density or timing	
		Over‐fixation	Was fixation too strong/long?	Reduce PFA concentration or time	
		Insufficient permeabilization	Intracellular target with mild buffer?	Use a stronger permeabilization buffer	
		Microwave power is too low	<100 W used?	Increase to 150–250 W	
		Temperature too low	<20°C during incubation?	Maintain 20–30°C	
Signal Strength	Weak signal	Low primary concentration	Only one dilution tested?	Test recommended ± higher/lower dilutions	Burry, 2011 Hewitt et al., 2014 Kumada et al., 2004 Muñoz et al., 2004 Chicoine & Webster, 1998 Galvez et al., 2006. Abcam (2024a) Lee & Kitaoka, 2018
		Short incubation	Insufficient ON/OFF microwave cycles?	Extend cycles	
		Thick culture	Multilayer or 3D sample?	Extend incubation; reduce power	
		Epitope masking	Excess fixation?	Reduce fixation strength	
		Low fluorophore brightness	Dim fluorophore used?	Switch to a brighter fluorophore	
Background / Noise	High background	Excess secondary antibody	Secondary too concentrated?	Increase dilution	Hewitt et al., 2014 Abcam, 2024b Burry, 2011 Giberson & Demaree, 1995 Smith et al., 2008 Kumada et al., 2004 Muñoz et al., 2004 Hajibagheri, 1999 Galvez et al., 2004
		Inadequate washing	Fewer than 3 washes?	Increase to 5 washes	
		Blocking serum mismatch	Serum species correct?	Match serum to secondary host	
		Autofluorescence	Over‐fixation?	Reduce fixation	
		Overheating	Temperature >30°C?	Reduce max temp; improve cooling	
		Uneven microwave field	No ColdSpot/water load?	Add standardized load	
		Continuous irradiation	No OFF cycles?	Use an intermittent program	
		Buffer contamination	Precipitation visible?	Filter fresh buffers	
Localization Errors	Diffuse staining	Over‐permeabilization	Excess Triton or detergent?	Reduce detergent exposure	Abcam, 2024b Hall, Hartwieg, and Nguyen, WormAtlas Lee & Kitaoka (2018) Giberson & Demaree, 1995 Smith et al., 2008
	Surface‐only staining	Under‐permeabilization	Intracellular target?	Increase permeabilization	
	Mislocalized organelle stain	Fixation incompatible with live stain?	Use 2% PFA for live‐stained samples		
	Junction protein mislocalization	Cells not confluent?	Increase seeding density		
	Structural distortion	Microwave overheating?	Maintain ≤37°C		
Uniformity	Patchy staining	Uneven cell seeding	Irregular density?	Standardize counting and plating	Lee & Kitaoka, 2018 Abcam, 2024b Giberson & Demaree, 1995 Josephsen et al., 2005 Chicoine & Webster, 1998 Muñoz et al., 2004
		Poor substrate coating	Attachment incomplete?	Optimize coating (PLL, ECM, etc.)	
		Heating heterogeneity	ColdSpot absent?	Add a load system	
		Variable cavity placement	Sample centered?	Fix consistent placement	
		Culture thickness variability	Multilayer clusters?	Normalize plating density	
Morphology	Cell shrinkage	Fixation too strong	>4% PFA used?	Reduce fixation	Abcam, 2024a Giberson & Demaree, 1995 Smith et al., 2008 Hajibagheri, 1999
	Membrane damage	Excess permeabilization	Strong detergent?	Reduce Triton exposure	
	Cytoplasmic distortion	Thermal stress	Temp >55°C reached?	Lower temperature limit	
	Osmotic imbalance	Buffer lacking sucrose?	Use an osmotic stabilizer		
Fluorescence Stability	Signal fading	Thermal quenching	Temp spikes?	Maintain ≤30°C	Boon & Kok, 1994 Muñoz et al., 2004 Kumada et al., 2004 Platt & Michael, 1985
	Photobleaching	Continuous microwave exposure	No OFF cycles?	Use ON/OFF cycling	
	Rapid fading	Mounting medium inadequate	Antifade present?	Use an antifade mounting medium	
Reproducibility	Run‐to‐run variability	Microwave not calibrated	Temperature probe validated?	Validate the probe regularly	Giberson & Demaree, 1995 Giberson et al., 1997 Giberson, 2002 Josephsen et al., 2005 Burry, 2011 Hewitt et al., 2014 Lee & Kitaoka, 2018
		Power drift	Nominal vs actual wattage confirmed?	Periodic output validation	
		Variable water load	Is the load volume consistent?	Standardize volume	
		Antibody lot change	New lot tested?	Re‐validate dilution	
		Passage variability	Passage range defined?	Standardize the passage number	
		Operator variation	Protocol deviations?	Standardize workflow	
3D / Organoids	Core negativity	Insufficient penetration	Thick matrix?	Extend cycles; reduce power	Chicoine & Webster, 1998 Muñoz et al., 2004 Galvez et al., 2006 Sanders et al., 2006 Giberson & Demaree, 1995
	Central damage	Power too high	Monolayer settings used?	Reduce power	
	Internal overheating	No OFF intervals	Continuous irradiation?	Increase OFF intervals	

### Understanding Results

An excellent tool for characterizing cell lines is ICC (Lee & Kitaoka, 2018). Positive immunoreactivity for a specific protein in a cell phenotype or cell line can confirm that cell lines are maintaining their characteristics (Burry, 2011; Hewitt et al., 2014). Although western blotting can be used for such confirmation (Burry, 2011; Hewitt et al., 2014), ICC clarifies whether a protein is correctly organized, expressed in the correct area, and associated with the particular organelle or cellular structure (Abcam, 2024b). Such clarification helps determine whether the characteristics of this cell line/phenotype are maintained.

**Figure 4:**
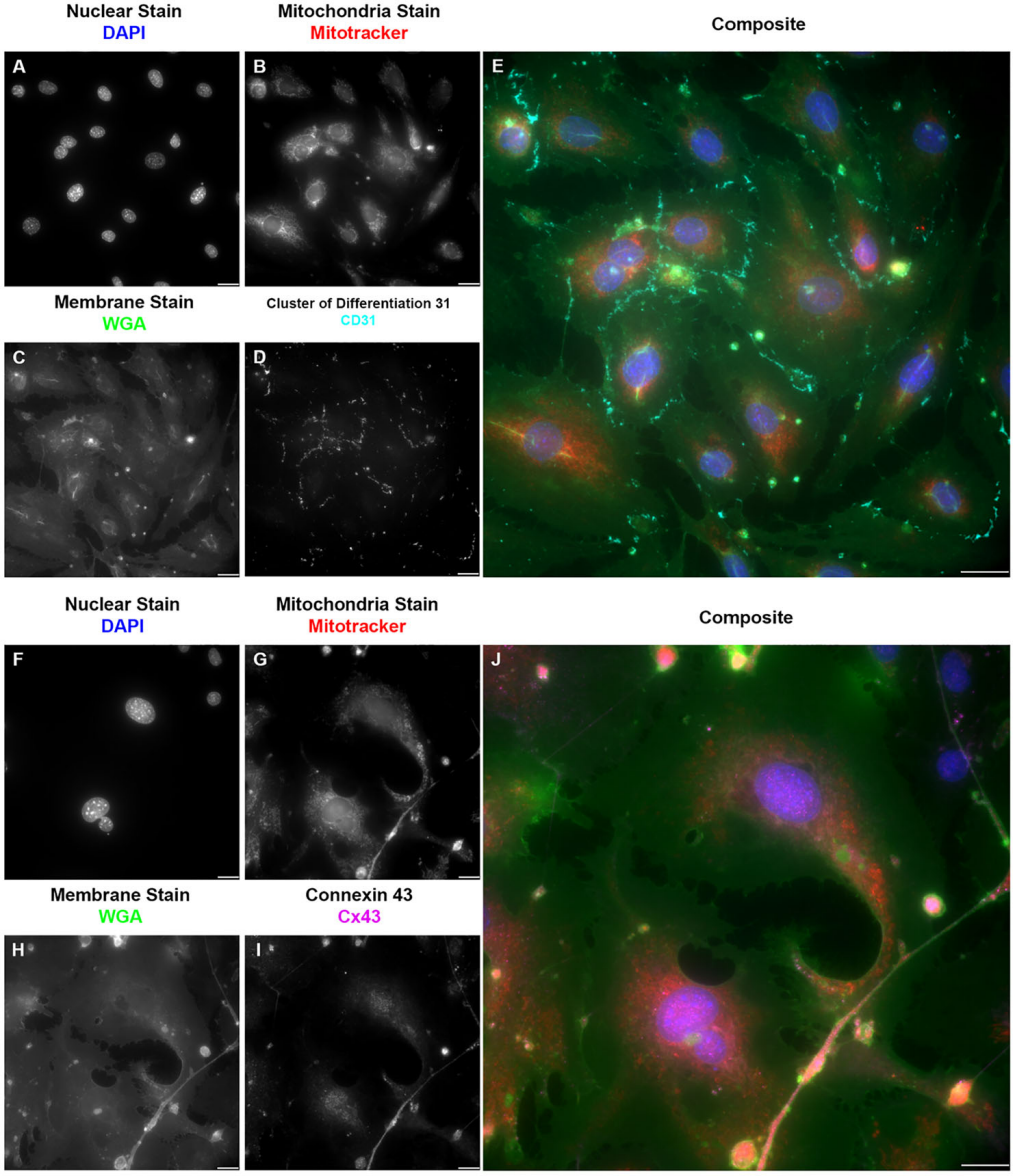
Comparison of microwave‐assisted ICC for endothelial cell marker CD31 and traditional ICC for endothelial cell marker Cx43 showed positive immunoreactivity for both labels, with reduced signal‐to‐noise ratio in microwave‐assisted ICC. (A/F) Grayscale image representation of the nuclear stain DAPI pseudo‐colored in blue on E and J. A lower background and a brighter signal are observed in the microwave‐assisted ICC (F‐J). (B/G) Grayscale image representation of the mitochondria stain, mitotracker, pseudo‐colored in red, on E and J. When B and G are compared, we see that microwave‐assisted ICC (A‐E) reduces the background (noise) in B, resulting in a brighter, more defined signal. (C/H) Grayscale image representation of the membrane stain wheat germ agglutinin (WGA), pseudo‐colored in green on E and J. This stain was used following permeabilization; this is evident from the labeling of other membrane organelles, which was also performed; nevertheless, the argument for a brighter signal and less noise with microwave‐assisted ICC remains valid for this stain. (D) Grayscale image representation of the endothelial cell marker cluster of differentiation 31 (CD31), also known as platelet endothelial cell adhesion molecule (PECAM‐1), pseudo‐colored in cyan in E. (E) Pseudo‐colored composite of traditional ICC against CD31 in endothelial cells. Images acquired at 60×, 0.9 NA; scale bar 20 µm.

**Figure 5:**
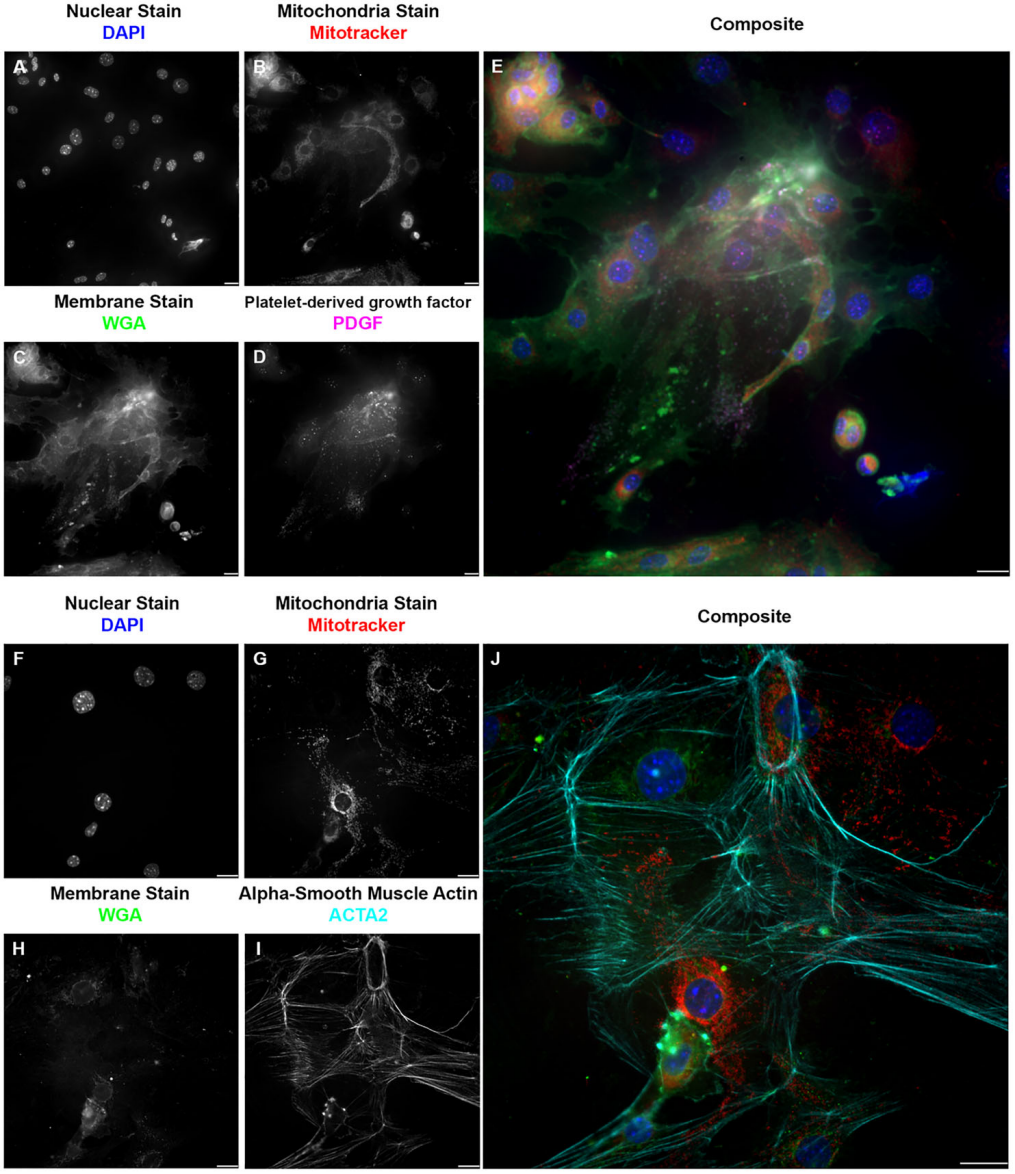
Comparison of traditional ICC for pericyte cell marker PDGFRB and microwave‐assisted ICC for pericyte cell marker ACTA2 showed positive immunoreactivity for both labels, with reduced signal‐to‐noise ratio in microwave‐assisted ICC. (A/F) Grayscale image representation of the nuclear stain DAPI pseudo‐colored in blue on E and J. (B/G) Grayscale image representation of the mitochondria stain Mitotracker pseudo‐colored in red on E and J. (C/H) Grayscale image representation of the membrane stain wheat germ agglutinin (WGA) pseudo‐colored in green on E and J. This stain was used following permeabilization; this is evident from the labeling of other membrane organelles. Nevertheless, the argument for a brighter signal and less noise with microwave‐assisted ICC remains valid for this stain. (D) Grayscale image representation of the pericytes marker platelet‐derived growth factor receptor (PDGFRB), pseudo‐colored in magenta in E. (E) Pseudo‐colored composite of traditional ICC against PDGFRB in pericytes. Images acquired at 40×, 0.60 NA; scale bar 20 µm. (I) Grayscale image representation of the pericyte marker alpha‐smooth muscle actin (ACTA2), pseudo‐colored in cyan on J. (J) Pseudo‐colored composite of microwave‐assisted ICC against ACTA2 in pericytes. Images acquired at 60×, 0.70 NA; scale bar 20 µm.

**Figure 6:**
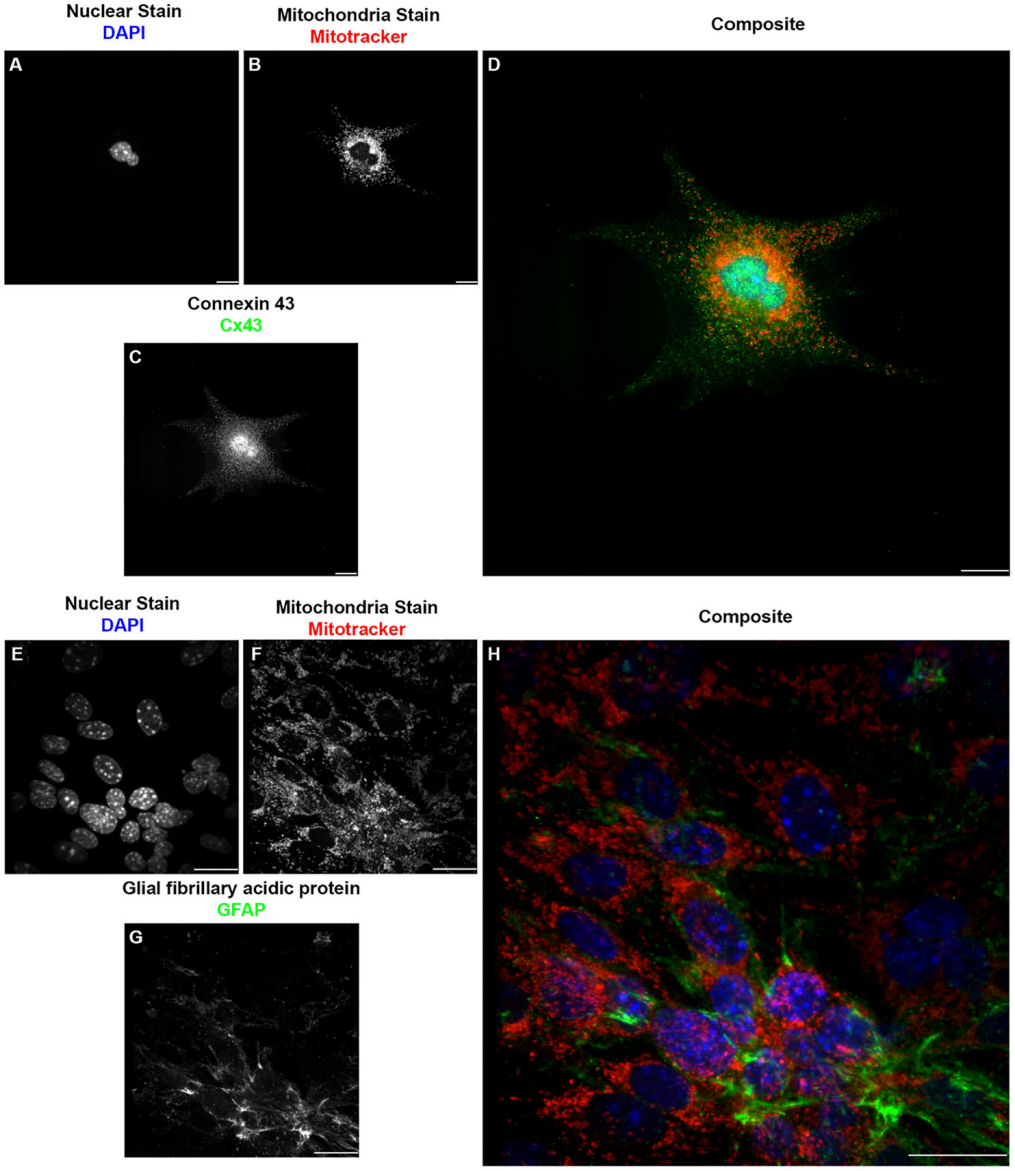
Comparison of microwave‐assisted ICC for astrocytic cell marker Cx43 and traditional ICC for astrocytic cell marker CGFAP showed positive immunoreactivity for both labels, with reduced signal‐to‐noise ratio in microwave‐assisted ICC. (A/E) Grayscale image representation of the nuclear stain DAPI pseudo‐colored in blue on D and H. (B/F) Grayscale image representation of the mitochondria stain Mitotracker pseudo‐colored in red on D and H. (C) Grayscale image representation of the astrocyte marker and gap junction protein connexin 43 (Cx43) pseudo‐colored in green on D. (D) Pseudo‐colored composite of microwave‐assisted ICC against Cx43 in astrocytes. Images acquired at 60×, 0.9 NA; scale bar 20 µm. (G) Grayscale image representation of the astrocyte marker glial fibrillary acidic protein (GFAP) pseudo‐colored in green on H. (H) Pseudo‐ colored composite of ICC against GFAP in astrocytes. Images are a project maximum Z, acquired at 60×, 1.42 NA oil, Step size 0.200 µm, Scale Bar 20 µm. In this figure, the reduction in noise, especially in the antibody labeling, occurs without changes in intensity or localization for the other markers.

**Figure 7:**
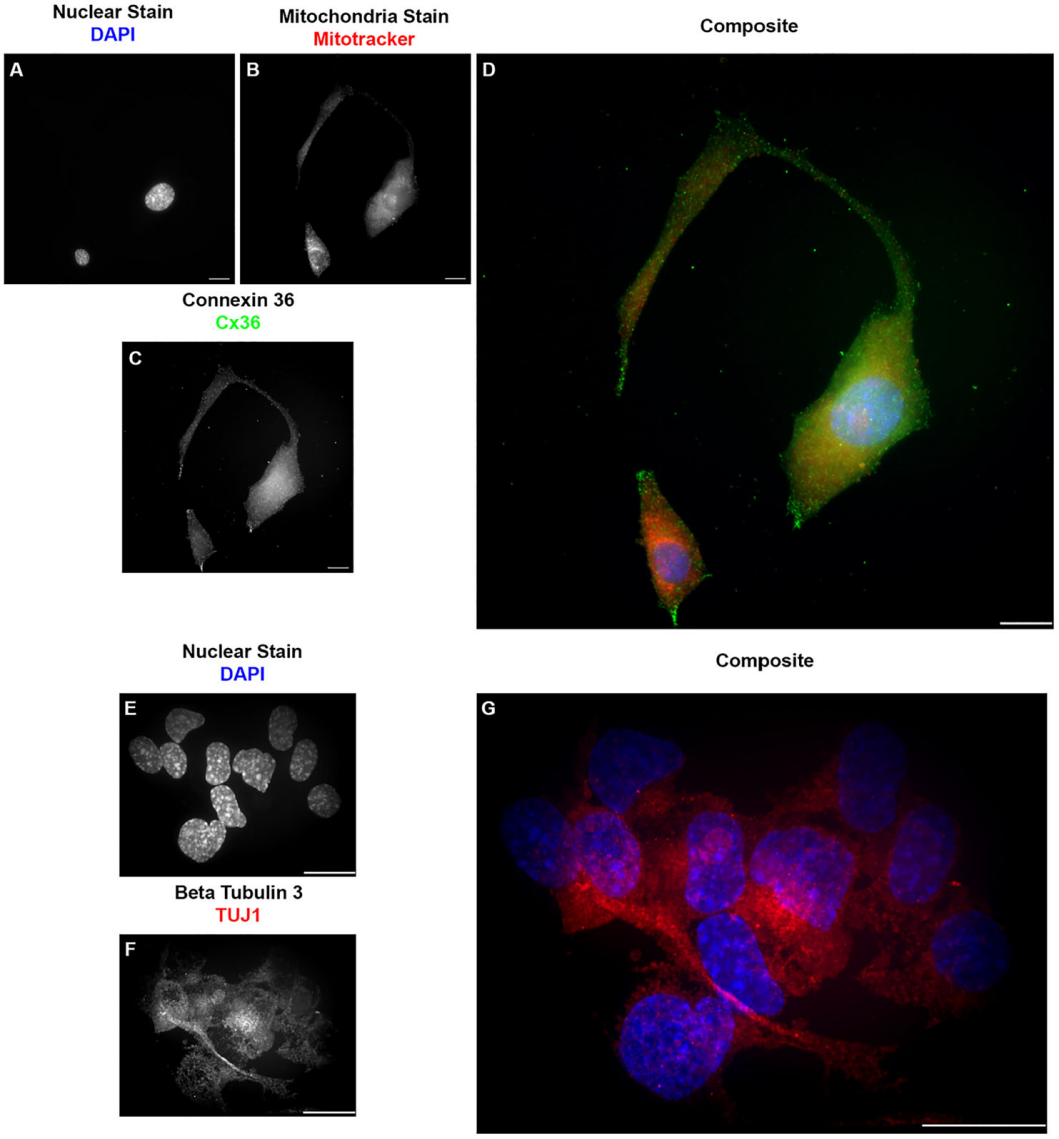
Comparison of microwave‐assisted ICC for motor neuron cell marker Cx36 and traditional ICC for motor neuron cell marker TUJ1 showed positive immunoreactivity for both labels, with reduced signal‐to‐noise ratio in microwave‐assisted ICC. (A/E) Grayscale image representation of the nuclear stain DAPI pseudo‐colored in blue on D and G. (B) Grayscale image representation of the mitochondria stains mitotracker pseudo‐colored in red on D. (C) Grayscale image representation of the motor neuron marker and gap junction protein connexin 36 (Cx36) pseudo‐colored in green on D. (D) Pseudo‐colored composite of microwave‐assisted ICC against Cx36 in motor neurons. Images acquired at 60×, 0.7 NA. Scale Bar: 20 µm. (F) Grayscale image representation of the motor neuron marker beta‐tubulin 3 (TUJ1) pseudo‐colored in red on G. (G) Pseudo‐colored composite of ICC against TUJ1 in motor neurons. Images are a project maximum Z, acquired at 100×, 1.40 NA oil, step size 0.250 µm, Scale Bar 20 µm. No changes in the intensity or localization of the selected markers are observed with microwave assistance.

Our research using ICC involves the cellular components of the blood‐spinal cord barrier, the counterpart of the blood‐ brain barrier in the spinal cord (Bartanusz et al., 2011; Jin et al., 2021). These cells are endothelial cells (specifically microvascular endothelial cells), pericytes, and astrocytes (Gong et al., 2025). We also added motor neurons to represent the components of the neurovascular unit (McConnell et al., 2017). For the endothelial cells (Figure 4), we used the commonly used marker, the cluster of differentiation 31 (CD31) (Figure 4 A‐E), also known as platelet endothelial cell adhesion molecule 1 (PECAM‐1) (Liu & Shi, 2012; Pisacane et al., 2007; Pusztaszeri et al., 2006; Vanchinathan et al., 2015; Wimmer et al., 2019). CD31, a transmembrane glycoprotein member of the immunoglobulin superfamily, is expressed in several cell phenotypes and is highly expressed in vascular endothelial cells (Liu & Shi, 2012; Pisacane et al., 2007; Pusztaszeri et al., 2006; Vanchinathan et al., 2015; Wimmer et al., 2019). CD31 is often used as a marker of blood–brain barrier integrity (Liu & Shi, 2012; Pisacane et al., 2007; Pusztaszeri et al., 2006; Vanchinathan et al., 2015; Wimmer et al., 2019). Another marker for endothelial cells is the gap junction protein connexin 43 (Cx43) (Figure 4 F‐J), a ubiquitous connexin found in the central nervous system in astrocytes and vascular endothelium (de Bock et al., 2011; Koepple et al., 2021; Okamoto et al., 2017; Yuan et al., 2015; Zhao et al., 2018). From the experiments reported here, we conclude that both markers have a positive immunoreactivity in endothelial cells. The use of the microwave as assistive technology for the ICC did not alter the expression or organization of these markers. For pericytes (Figure 5), we used known pericyte markers, platelet‐derived growth factor receptor beta (PDGFRB) (Figure 5 A‐E), receptors found only in pericytes (Bondjers et al., 2006; Niu et al., 2014; Smyth et al., 2022; Smyth et al., 2018; Winkler et al., 2010), and the alpha‐smooth muscle actin (ACTA2) (Figure 5 F‐J) (Kim et al., 2020). For astrocytes (Figure 6), we used their known standard markers, ubiquitous connexin 43 (Figure 6 A‐D) (Basu & Das Sarma, 2018; Boulay et al., 2018; Fatemi et al., 2008; Yin et al., 2018) and the glial fibrillary acidic protein (GFAP) (Figure 6 E‐H) (Benedet et al., 2021; Dolman et al., 2004; Jones et al., 2018; Kamphuis et al., 2012). As with endothelial cells and pericytes, we show that the markers are positively immunoreactive in astrocytes. The use of the microwave as an assisting technology for the ICC did not alter the expression, pattern, or organization of these markers. For motor neurons (Figure 7), we used the neuronal connexin, connexin 36 (Figure 7 A‐D) (Bautista et al., 2014; Belousov et al., 2018; Hartfield et al., 2011), and the neuronal marker beta‐tubulin 3 (TUJ1) (Figure 7 E‐G) (Latremoliere et al., 2018; Lee et al., 2020; Qu et al., 2014). As with endothelial cells, pericytes, and astrocytes, we showed that markers exhibit positive immunoreactivity in motor neurons. As in our prior results, the use of the microwave as assistive technology did not alter the expression or organization of these markers.

In conclusion, we demonstrate that microwave‐assisted ICC is an efficient technique that reduces background signal (noise) without damaging cellular antigens. The technique allowed characterization of the components of the BSCB/neurovascular unit. The BSCB/neurovascular unit components showed positive immunoreactivity for specific cell phenotype markers: endothelial cells (CD31 and Cx43), pericytes (PDGFRB and ACTA2), astrocytes (Cx43 and GFAP), and motor neurons (Cx36 and TUJ1). Live organelle stains, such as the mitochondrial marker MitoTracker, were neither altered, weakened, nor lost after microwave‐assisted ICC; the same was true for other organelle stains, such as DAPI and WGA. These stains were used as reference or “landmarks” to assess the accuracy and the spatial distribution of the proteins of interest. Nevertheless, it is important to note that following permeabilization, the WGA signal‐to‐noise ratio differs from that in non‐permeabilized samples (data not presented here), and that proper fixation protocols for ICC and IHC with paraformaldehyde did not completely crosslink the stain. This led to the difference in signal‐to‐noise noticed across figures (Abcam, 2024a). Microwave‐assisted ICC is a time‐consuming and cost‐effective technique that provides consistent results (Muñoz et al., 2004).

### Time Considerations

The total time needed will depend on the protocols followed. For example, the alternative protocol to Basic Protocol 2 tends to be longer. However, most of the time will be spent culturing cells. The time spent characterizing cells depends on the chosen protocol. Below are approximate time estimates for each protocol to aid in planning.

#### Basic Protocol 1: Culture of cellular components of the Blood–Spinal Cord Barrier and Neurovascular Unit

- Initial thawing and recovery: 1–2 days
- Routine culture to reach 70–80% confluency: 2–5 days (cell‐type dependent)
- Trypsinization, counting, and reseeding: ∼1–1.5 hr
- Preparation of low‐passage frozen stocks: ∼1 hr (excluding freezing time)
- Seeding cells on 8‐well imaging slides: ∼30 min
- Cell attachment and growth prior to ICC: Minimum 16 hr (typically overnight)

*Total active hands‐on time: ∼3–4 hr*

*Total elapsed time (including growth and incubation): ∼3–7 days*

#### Basic Protocol 2: Microwave‐assisted immunocytochemistry (PELCO BioWave® Pro)

##### Microwave‐Assisted Steps (Table 4)

The cumulative microwave run time for Steps 1–20 is ∼53 min, calculated as follows:

- Rinses (HBSS/HBSS:Su/HBSS:Su:Sap): ∼9 min
- Blocking (ON/OFF cycles): ∼3 min
- Primary antibody incubation (ON/OFF/ON): ∼16 min
- Secondary antibody incubation (ON/OFF/ON): ∼15 min
- Organelle stain (ON/OFF/ON): ∼11 min

*Total BioWave® Pro processing time: ∼53 min*

Additional Non‐Microwave Steps

- Fixation with 2% PFA: 10 min
- Final mounting (Vectashield): ∼10–15 min

*Total active hands‐on time: ∼1–1.25 hr*

*Total elapsed time (from fixation to mounted slide): ∼1.5 hr*

With this protocol, you can fix, stain, and image your samples in a single day. This significantly reduces processing time compared to conventional ICC.

#### Alternate Protocol 1: Conventional Immunocytochemistry

- Fixation: 10 min
- Wash steps: ∼15–20 min
- Permeabilization: ∼15 min
- Blocking: 30 min
- Primary antibody incubation:
  ○ 2 hr at room temperature or
  ○ Overnight (12–16 hr) at 4°C
- Wash steps: ∼15–20 min
- Secondary antibody incubation: 1 hr
- Wash steps: ∼15–20 min
- Organelle staining: ∼30 min (plus optional live‐stain incubation prior to fixation)
- Final washes and mounting: ∼15 min
*Total active hands‐on time: ∼2.5–3.5 hr*
*Total elapsed time*:
  ○ ∼6–7 hr (room‐temperature primary incubation)
  ○ ∼18–24 hr (overnight primary incubation)

### Summary

- **Cell** culture requires the most time overall, since it depends on the cells' biology.
- **Microwave‐assisted ICC** lets you process your samples quickly—about 53 min of processing time and a total elapsed time of around 1.5 hr—so you can get to the microscope sooner.
- **Conventional ICC** usually requires overnight or a full‐day incubation. Microwave‐assisted ICC is best when you need fast, reproducible results without compromising antigen integrity or imaging quality.

Although a full statistical comparison was not the primary objective of this study, qualitative and semi‐quantitative observations from replicate experiments highlight clear procedural and efficiency differences between conventional and microwave‐assisted ICC (Table 5)

**Table 5:** Qualitative performance comparison between conventional and microwave‐assisted ICC

Metric	Conventional ICC	Microwave‐Assisted ICC
Total elapsed time (fixation to mounting)	∼6–24 hr (depending on primary incubation)	∼1.5 hr
Active hands‐on time	∼2.5–3.5 hr	∼1–1.25 hr
Primary antibody incubation	2 hr RT or overnight at 4°C	∼16 min (ON/OFF cycling)
Secondary antibody incubation	1 hr	∼15 min
Reagent diffusion rate	Passive diffusion	Accelerated diffusion via dielectric heating
Antibody penetration (monolayer cells)	Adequate	Enhanced and more uniform
Background staining	Moderate; dependent on incubation length	Reduced due to shortened exposure time
Signal‐to‐noise ratio	Good with optimization	Improved in tested conditions
Reagent consumption	Standard volumes	Reduced effective exposure time; lower reagent demand
Replicate variability	Dependent on operator timing	Reduced temporal variability with programmed cycles
Equipment requirement	Standard laboratory supplies	Requires a calibrated microwave system

### Author Contributions

**Aidyn M. Medina‐López**: Writing—original draft; writing—review and editing; conceptualization; investigation; methodology; validation; visualization; software; formal analysis; data curation. **Irma I. Torres‐Vázquez**: Resources; validation; writing—review and editing; methodology; supervision. **Noraida Martínez‐Rivera**: Supervision; resources; data curation; visualization; writing—review and editing; methodology; software; formal analysis; project administration. **Eduardo Rosa‐Molinar**: Investigation; conceptualization; funding acquisition; writing—review and editing; methodology; supervision; resources; data curation; project administration; formal analysis.

### Conflict of Interest

The authors have no conflicts of interest to declare.

## Data Availability

The data that support the findings of this study are available from the corresponding author upon reasonable request.
